# Scoping Review of Treatment Strategies for Holmes Tremor: Pharmacological and Surgical Interventions

**DOI:** 10.5334/tohm.1019

**Published:** 2025-06-17

**Authors:** Gabriel Chung, Henrique Ballalai Ferraz

**Affiliations:** 1 Department of Neurology, Movement Disorders Unit, Hospital São Paulo, Escola Paulista de Medicina, Universidade Federal de São Paulo, São Paulo City, Brazil

**Keywords:** Holmes Tremor, Treatment, Pharmacology, Surgical Intervention, Review

## Abstract

**Background:**

Holmes tremor is a rare neurological condition, first described in 1904, characterized by a low-frequency tremor that manifests at rest, posture, and action. Despite its recognition for over a century, effective treatment strategies for Holmes tremor remain elusive due to its rarity and challenges in conducting robust studies.

**Methods:**

Given that the existent medical literature on Holmes tremor is based on reports and case series, we conducted a survey using the keywords “Holmes tremor” and “Rubral tremor” to analyze the therapeutic approaches utilized, as well as their success rate.

**Results:**

We have found 121 Holmes tremor patients across 97 publications. Levodopa and anticholinergics emerged as the most common employed pharmacological treatments, demonstrating significant response rates. Dopaminergic agonists are also promising therapeutic approaches, albeit with fewer reported cases. From the surgical perspective, functional neurosurgery offers substantial benefits to the symptomatic control of patients, with deep brain stimulation electrodes being a promising strategy.

**Discussion:**

Pharmacological treatment, specifically levodopa, anticholinergics and dopaminergic agonists exhibit the highest success rate in managing Holmes tremor. Additionally, surgical strategies may help tremor control as supported by previous reviews with quantitative analysis. However, the heterogeneity in the reporting of these cases underscores the need for standardized case descriptions to permit conclusions.

**Highlights:**

This study brings a new perspective to the treatment of HT, as it estimates which medications and interventions may be more consistent for the treatment for Holmes Tremor.

## 1. Introduction

Holmes Tremor (HT) was first described by Gordon Holmes in 1904 [1,2], and is currently defined as a resting, postural, and intention tremor that usually results from the contraction of both proximal and distal muscles, occurring at a frequency of less than 5 Hz [3]. Historically, HT has been known by other names, including rubral, thalamic and midbrain tremor, but the continuous observation of this phenomenon soon demonstrated that a lesion in these structures was not strictly necessary [1].

It is our understanding today that, from a pathophysiological point of view, HT usually manifests a few months after the onset of a lesions in both the nigrostriatal, cerebellothalamic-cortical, and dentate-rubro-thalamic pathways [4]. Etiologically, HT is most associated with vascular or traumatic lesions, but many other causes have been described [1].

The treatment of HT remains challenging even in specialized services, and inadequate symptom control can lead to a significant loss of functionality for affected patients [1]. Given the rarity of the condition, current evidence is primarily derived from case reports, case series and expert opinions; therefore, it is essential to systematically structure and organize the data collected to build a robust body of knowledge about HT.

## 2. Methodology

A review of the literature was carried out using articles indexed in the Pubmed database. A search was performed with the keywords “Holmes tremor” and “Rubral tremor”, including studies written in English on case reports, case series, case-control and clinical trials, without any period filter. Review and meta-analysis studies were excluded either through platform filters or manually. In the case of “Rubral tremor” search, an additional filter was applied to select only articles that include the keyword in its title.

The studies were first analyzed based on their abstract and subsequently reviewed in full. We included articles that defined Holmes Tremor based on Holmes’ original description [2], or Movement Disorders Society’s diagnosis criteria, published in 1998 [5] and again in 2018 [3]. Articles suggesting alternative diagnoses or involved with comorbidities that could potentially interfere with the treatment response were excluded. Articles that did not address the treatment or did not have sufficient data on individual responses to the established therapeutic strategies were also excluded.

The articles were organized into a table for categorization according to the treatment modality (pharmacological or surgical) with results classified as positive (complete or partial) and negative (absent or minimal). The drugs used were listed along their outcomes, and when formal quantifications by means of scales were available, they were also recorded. In the case of surgical treatment, the modality and surgical target were described.

## 3. Results

**Figure 1:**
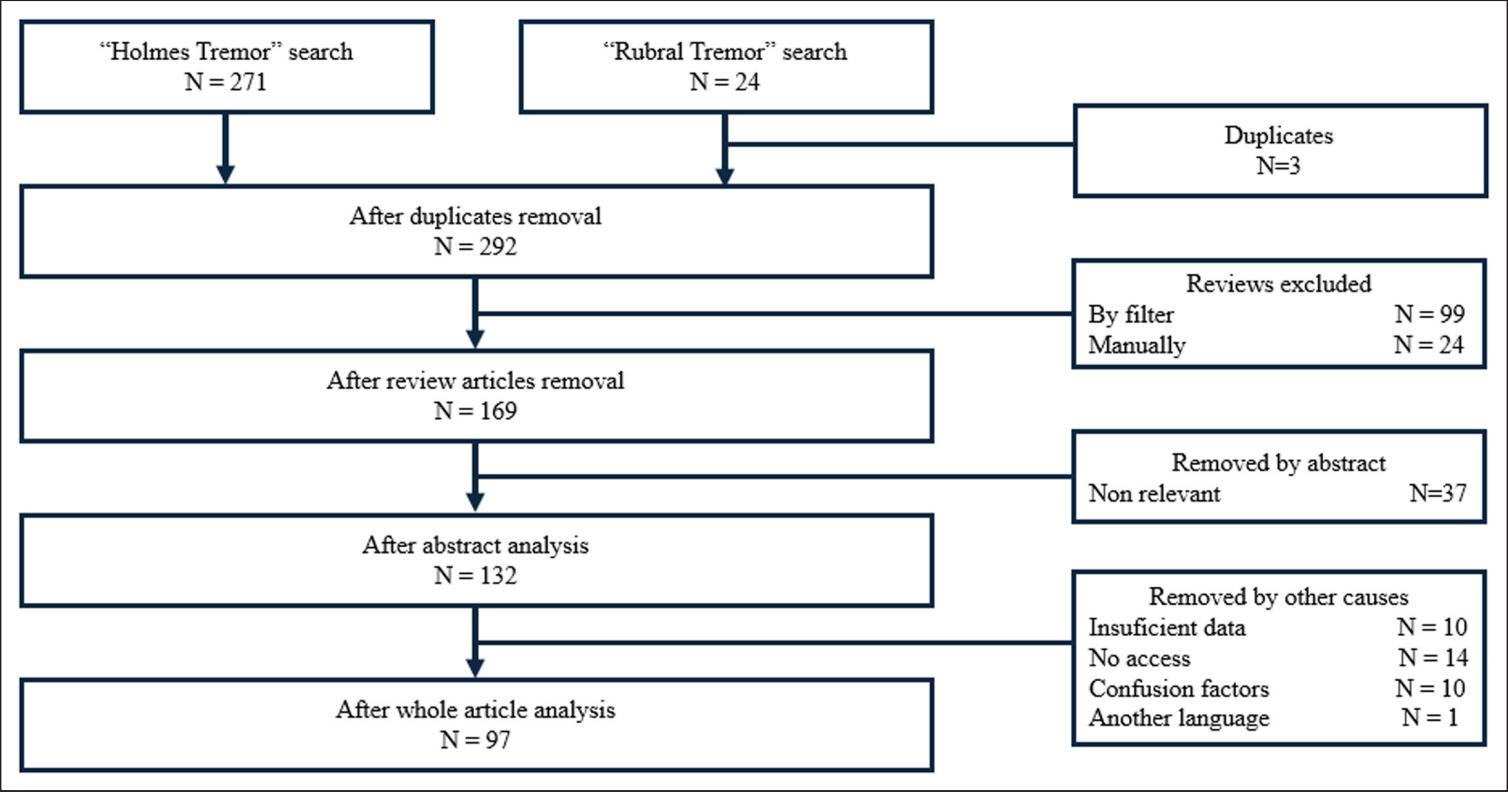
Article selection flowchart.

From the search with the established keyword, 295 articles published from 1927 to 2025 were found. Article selection process detailed in Figure 1 led to the exclusion of 198 articles. The remaining 97 publications consist of reports and case series, accounting for 121 cases.

Among the cases reviewed, 15 did not respond to treatment [6,7,8,9,10,11,12,13,14,15,16,17,18,19,20] and, in one case, treatment was deemed unnecessary due to the absence of any impact on patient’s quality of life [6]. Of the remaining cases, 42 showed positive results with pharmacological therapies either as mono or polytherapy [6,21,22,23,24,25,26,27,28,29,30,31,32,33,34,35,36,37,38,39,40,41,42,43,44,45,46,47,48,49,50,51,52,53] and another 41 with surgical therapies [6,7,39,54,55,56,57,58,59,60,61,62,63,64,65,66,67,68,69,70,71,72,73,74,75,76,77,78,79,80,81]. Furthermore, 14 cases responded to treatment of the underlying disease leading to HT [46,82,83,84,85,86,87,88,89,90,91,92,93,94] while 5 cases achieved positive outcomes through combined treatment of the underlying disease with an additional therapy (drug or surgical) [95,96,97,98,99]. There are also four reports of transient HT [100,101,102] and one with improvement following acupuncture [44]. One case presented a satisfactory response to drug therapy, but due to adverse effects of the medication, a subsequent surgical intervention was performed. For the purpose of assessing the response to treatment, the case was included in both therapeutic categories [39].

HT reversed by treatment of the underlying disease, included: pineal hamartoma [90], secondary to antipsychotic use [83], paraneoplasm [84], cavernoma [87,88,89], germinoma [86], nonketotic hyperglycemia [92], arachnoid cyst [46,94], multiple myeloma [96]aneurysm [85], neuroinfection [97,99], tumefactive perivascular space [82], CSF hypotension [91] and hydrocephalus [98].

### 3.1. Pharmacological treatment

#### 3.1.1. Levodopa

Levodopa was the most used therapy in all the studies analyzed, being described in 67 cases [6,7,9,11,14,15,17,18,19,21,22,23,25,28,29,31,32,33,34,35,36,38,39,40,41,43,44,47,49,50,52,53,55,56,59,60,61,64,66,67,69,70,71,72,73,76,77,78,80,89,93,95,96,98,101]. Twenty-one patients (31.34%) showed partial or satisfactory improvement [6,22,23,28,29,33,35,39,40,41,47,50,52,53,98]. The minimum daily dose was 150 mg and the maximum was 1500 mg, with a median of 600 mg. Only two studies evaluated the response by rating scales. The magnitude of response, measured by UPDRS-III were 57% and 70% [33,39].

Regarding positive outcomes, multiple drugs were required in 7 [23,29,33,47,98] of the 21 cases, with dopamine agonists being the main combination with 4 reports, followed by levetiracetam and isoniazid, both with one; one article reported positive results with the combination of levodopa, pramipexol and zonisamide. In these cases, some authors highlight the preferential effectiveness of levodopa for the resting component of HT [23,33]. Additionally, there are notable records of improvement with single doses of the medication [50,53]. As for the 46 negative outcome cases, only 37 developed to successful treatments, 22 of which were surgery referrals.

#### 3.1.2. Dopaminergic agonists

Eleven dopamine agonists cases were analyzed [21,24,25,27,29,32,33,45,47,67,99]. Ten had positive outcomes, being 5 with pramipexole [27,29,33,45,99], 3 with piribedil [32,47] and 3 isolated reports with ropinirole [21], bromocriptine [47] and cabergoline [25]. Bromocriptine prescription was ineffective in two cases [24,67]. The daily doses of pramipexole ranged between 1.08 and 2.8 mg, while those of piribedil ranged between 120 and 300 mg [27,29,32,33,45,47,99].

Of the 10 reports with positive responses, only 4 occurred with the isolated use of dopamine agonists, whereas, 5 had to combine levodopa to achieve satisfactory results [29,33,47] and one combined pramipexole, levodopa and zonisamide. Some reports show positive outcomes even in the absence of response to levodopa [21,25,32], and one report described a successful treatment with ropinirole in an absent response to pramipexole [21]. The case report in which dopamine agonist didn’t show any benefit was referred to surgery [67].

#### 3.1.3. Primidone

The use of primidone has been reported in 13 cases [6,7,14,34,56,59,60,70,71,96,101], with only one case of partial improvement at the daily dose of 250 mg [6]. Among the cases with negative outcomes, one showed improvement with isoniazid [34], another exhibited spontaneous improvement [101], 4 underwent surgical treatment [56,59,60,70,71,96] and the remaining cases showed no response.

#### 3.1.4. Beta blockers

A total of 17 cases reported the use of beta blockers [7,13,14,17,26,36,37,56,58,59,70,71,73,75,77,79,96], of which 14 involved propranolol. None of them achieved a satisfactory response; ten proceeded to surgical therapy [56,58,59,70,71,73,75,77,79,96] and 3 responded to other treatments [26,36,37].

#### 3.1.5. Anticholinergics

Anticholinergics were the second most frequently used drug class in the studies reviewed, being described in 24 cases [13,14,17,22,23,24,30,31,34,37,48,51,52,64,66,70,77,78,78,80,93,95,97]. Of these, 7 (29.16%) demonstrated positive results [22,24,30,37,48,51,97], with only 4 cases utilizing anticholinergic monotherapy [24,37,48,51]. Two of the seven warrant special consideration: one had associated mild dystonic features [30]; while the other received concurrent treatment for a neuroinfection causing HT [97]. Among the cases with negative results, 9 underwent surgical and four responded to other medication treatment [64,66,70,77,78,78,80,95].

The most successful drug was trihexiphenidyl, with a daily doses ranging from 6 to 38 mg [30,48,51,97], with a median of 12.5 mg; of the four reports with positive trihexiphenidyl results, one used combined therapy with amantadine and one with clonazepam. Other medications with satisfactory results were benztropine and biperiden [22,24,37].

#### 3.1.6. Anti-seizure medication

Anti-seizure drugs showed satisfactory response in only 7 cases, with 22 negative results. Levetiracetam was successful in three [26,31,98] out of a total of 14 attempts [7,12,13,15,26,31,32,38,59,61,61,67,98]; valproic acid in 1 of 5 [22,38,49,51,73], only in combination with benztropine; topiramate in 1 of 6, in monotherapy [7,38,55,56,70,96]; and zonisamide in 2 of 6 [33,42,56,60,60,84] attempts, one in monotherapy and one in combination with levodopa and pramipexole. Gabapentin had 7 negative attempts [6,13,15,38,59,96].

Regarding the use of zonisamide, some authors described that its use in association with dopaminergic drugs was interesting because it had the best effect for the controlling posture and intention tremor within the combinations used [6,13,15,38,59,96].

#### 3.1.7. Benzodiazepines

Although they are the second most reported class, with a total of 36 cases [7,13,14,15,17,19,20,22,23,26,28,31,34,35,43,44,48,49,51,55,60,66,69,73,75,79,80,84,93,95,96,97,98,101], benzodiazepines presented positive results only in four cases (11.11%) [43,44,49,97] with the clonazepam being the most prescribed, in doses of up to 3 mg per day, 3 of which were in monotherapy [43,44,49], and one in combination with trixiphenidyl.

#### 3.1.8. Botulinum toxin

Of the five cases reviewed involving the use of botulinum toxin [36,61,73,79], only one demonstrated improvement as measured by the The Essential Tremor Rating Assessment Scale (TETRAS), with a response rate of 37.77% [36]. In this particular case, the patient failed to respond to levodopa, propranolol and clonazepam; the injections scheme was made with abobotulinotoxin A in the following muscles: 500 IU in the biceps brachii, 250 IU in the triceps brachii and 250 IU in the deltoid [36].

#### 3.1.9. Other drugs

There are two successful reports with isoniazid in HT patients caused by neurotoxoplasmosis [23,34]. In addition, amantadine in combination with triexiphenidyl produced a positive result in one case [30].

### 3.2. Surgical Treatment

#### 3.2.1. Lesion surgery

We identified 8 reports of lesional surgeries for the treatment of HT [57,58,63,69,73,77,95,96], one of which was performed after the removal of the deep brain stimulation electrode due to infection [57]. Among these, 6 have satisfactory symptom control, with surgical targeting placed either in the internal Pallidus Globe (GPi) [69] or Ventrous Intermediate Nucleus of the Thalamus (VIM) [57,63,73,95,96]. Notably, only the GPi lesion patient was evaluated using the Tremor Rating Scale, with a rate of response of 54%.

Of the remaining cases, one showed an unsatisfactory outcome following a unilateral VIM lesion with high-frequency ultrasonography [58], while another experienced symptoms recurrence after stereotactic VIM lesion with transient symptom improvement [77].

#### 3.2.2. Neuromodulation

Neuromodulation surgery demonstrated the highest consistency among the treatments evaluated in this review, with positive results reported across all the reports surveyed, including complete improvements measured by rating scales. Among 40 cases surveyed, 25 involved single-target stimulation while 15 utilized multi-target stimulation, as shown in Table 1.

**Table 1:** Summary of the cases whose treatment was performed with neuromodulation with deep brain stimulation. The number of patients in each case report or series, gender and age of the patients, laterality of the surgery, scale used for evaluation, and response rate with treatment are described. N number of patients who underwent DBS implantation in each series; M Male; F Female; ND Not described.; GPi: Internal globus pallidus; VIM: Ventral intermediate nucleos of the thalamus; Vo: Ventro-oral nucleous of the thalamus; Voa: Ventro-oral anterior nucleous of the thalamus; Vop: Ventro-oral posterior nucleous of the thalamus; ZI: Zona incerta; PSA: Posterior subthalamic area; DRTT: Dentato rubro thalamic tract; RaPrl: Prelemniscal radiations; TETRAS: The Tremor Research Group Essential Tremor Rating Scale.


	N	SEX	AGE	LATERALITY	TARGET	SCALE	RATE OF IMPROVEMENT

Del Gaudio N, et al [6]	1	M	18	Unilateral	VIM+GPi	Fahn Tolosa Marin	67%

Rosa TD, et al [56]	1	M	58	Bilateral	VIM+PSA	Fahn Tolosa Marin	29,26%

Razmkon A, et al [57]	1	M	37	Unilateral	VIM		

O’Shea SA, et al [39]	1	F	62	Unilateral	VIM+ZI	UPDRS III	33,33%

Brittain JS, et al [72]	1	ND	36	Unilateral	Vop+ZI	Bain Rating Scale	Rest 89%Postural 90% Intention 32%

Yousefi O, et al [81]	1	M	57	Unilateral	PSA		

Maesawa S, et al [58]	1	F	77	Unilateral	VIM+RaPrlVa+ZI	Clinical Rating Scale for Tremor	34,78%

Ghanchi H, et al [59]	1	M	66	Unilateral	VIM	TETRAS	75%

Kamo H, et al [60]	2	M	18	Unilateral	PSA	Fahn Tolosa Marin	56,45%

		M	52	Bilateral	PSA	Fahn Tolosa Marin	33,92%

Bargiotas P, et al [61]	4	F	62	Unilateral	VIM	Fahn Tolosa Marin	4,50%

		M	71	Unilateral	DRTT	Fahn Tolosa Marin	7,90%

		F	28	Unilateral	DRTT	Fahn Tolosa Marin	22,70%

		M	46	Unilateral	VIM	Fahn Tolosa Marin	33,30%

Onder H, et al [55]	1	M	58	Unilateral	VIM	Fahn Tolosa Marin	73%

Kobayashi K, et al [62]	4	F	19	Unilateral	VIM+PSA	Fahn Tolosa Marin	100%

		M	67	Unilateral	VIM+PSA	Fahn Tolosa Marin	100%

		M	44	Unilateral	VIM+PSA	Fahn Tolosa Marin	95%

		M	18	Unilateral	VIM+PSA	Fahn Tolosa Marin	93%

Kilbane C, et al [74]	4	M	43	Unilateral	VIM+Voa+GPi		

		M	65	Unilateral	GPi		

		M	29	Unilateral	VIM+GPi		

		M	50	Unilateral	GPi		

Chong Z, et al [64]	1	M	53	Unilateral	VIM+PSA	Fahn Tolosa Marin	62,50%

Toda H, et al [75]	1	M	16	Unilateral	Vo+PSA	Fahn Tolosa Marin	71,73%

Peker S, et al [65]	1	F	11	Unilateral	VIM		

Castrop F, et al [78]	2	ND	43	Unilateral	VIM		

		ND	40	Unilateral	VIM		

Aquino CC, et al [96]	1	M	66	Unilateral	VIM		

Grabska N, et al [66]	1	F	45	Unilateral	Vo+ZI	Fahn Tolosa Marin	73,80%

Acar G, et al [67]	1	M	31	Bilateral	VIM		

Plaha P, et al [54]	1	M	84	Bilateral	ZI	Fahn Tolosa Marin	70,20%

Romanelli P, et al [71]	1	M	79	Unilateral	VIM		

Hertel F, et al [68]	1	M	58	Unilateral	VIM		

Foote KD, et al [76]	1	M	24	Unilateral	VIM Voa+Vop	Tremor Rating Scale	39,62%

Nikkhah G, et al [79]	2	F	47	Unilateral	VIM		

		F	37	Unilateral	VIM		

Kudo M, et al [80]	1	F	67	Bilateral	VIM		

Goto S, et al [69]	1	M	53	Unilateral	VIM	Tremor Rating Scale	45,45%

Sanborn MR, et al [70]	1	M	31	Unilateral	VIM		


Some noteworthy observations were reported: of improvement were documented in two cases even after device shutdown, and bilateral surgeries were suggested to provide additional improvement even in a case of unilateral symptoms as reported by Kudo et al [80].

## 4. Discussion

This review includes a considerable number of HT cases, bringing a new perspective on its treatment. By analyzing the enrolled studies, we can estimate the rate of success of different treatments, which will eventually help in therapeutic decision-making and guide future research on HT.

However, this is not the first attempt to summarize HT treatment strategies. Wang et al. [103] published a systematic review containing quantitative tremor response rates to different treatments, and by analyzing the effects of individual drugs, they were unable to determine any superiority, but they also had a limited sample size. Interestingly, they reported levetiracetam as a promising drug for tremor control [103], but our data suggest it can be useful only in a few cases, since levodopa, trihexyphenidyl, and even dopaminergic agonists have shown better response rates. When it comes to surgical intervention, there are better symptomatic control with neuromodulation as compared to pharmacological treatment alone [103]. Even though our study was not designed to compare the efficacy of treatments, the response rate of HT to neuromodulation was indeed remarkable, making it a viable option for HT patients refractory to medication.

Success rates are not widely discussed in current medical literature, possibly due to both the scarce number of HT cases and the limited access to large cohorts. Accordingly, current treatment is based on case reports findings. Nonetheless, Raina et al. [4] published an impressive case series with 29 patients and a 54.16% success rate with levodopa treatment, and a similar median daily dosage as reported in our study. Formal quantitative measurements were not performed by their team, but they reported almost complete tremor control with levodopa use in half of the responsive patients [4]. As for other pharmacological agents, a limited number of successful cases were described with botulinum toxin, but no satisfactory results were described with trihexyphenidyl, levetiracetam, clonazepam, propranolol, or other antiepileptic drugs [4]. Given that our review is based on published clinical cases, it is possible that the benefits of levodopa are overestimated, as patients who did not respond to the proposed treatment are less likely to be subjects of scientific publication. For the same reason, it may also overestimate the success rates of other treatments, both pharmacological and surgical, and the actual rate of success could be lower.

**Figure 2:**
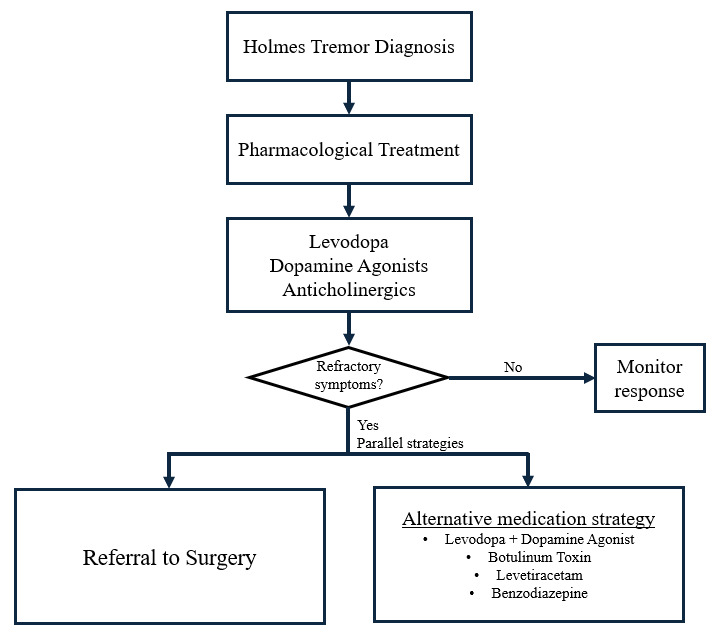
Proposed algorithm for Holmes Tremor treatment.

Based on the gathered data, we propose levodopa, trihexyphenidyl and dopamine agonists as first line medication for HT treatment, having the combination of levodopa and dopamine agonists as a reasonable combination in refractory cases. Surgical interventions are not yet investigated as first line options, but, as it seems to be the most successful strategy, caregivers should not delay referral in medication resistant HT. The summarized strategies for HT treatment based on our findings can be seen in Figure 2.

Our study brings a new perspective to the treatment of HT, as it estimates which medications may be more consistent for HT patients. Because HT is rare, little systematized information is available, and we believe our review is a crucial step in HT research and in clinical practice. For the future, we propose systematizing HT cases and series reports, including failed treatment attempts, medication dosages, and quantitative assessments of HT severity before and after each attempt; additionally, description of associated phenomena, such as dystonia, parkinsonism and ataxia, should be reported and measured as well. Additionally, HT literature itself is dubious as its diagnosis is clinical and eventually other entities could mimic it for untrained professionals, specially due to the rarity of this kind of tremor; the lack of tools to objectively evaluate HT in particular also play a role in the practical difficulties in its assessment Of course, our study has other limitations, mainly associated with publication bias and incomplete information in published papers, but it sheds light on the current HT approach since there is an absence of clinical trials. Large case series represent a viable approach to building knowledge regarding the true response to treatments, as the greater the number of patients treated, the higher the likelihood of observing both positive and negative outcomes within the same sample.

The present review indicates that the drug treatments with the highest response rate are levodopa, anticholinergic drugs and dopamine agonists. From a surgical point of view, neuromodulation with DBS implantation has positive results, regardless of the target selected among those described. However, being a review based on reports and case series, larger conclusions cannot be made reliably due to bias; the heterogeneity of the data in the reports and in the treatments instituted, as well as the application of different scales are some of the impediments to detailed analysis. This review should, therefore, not be seen as a definite guideline on HT treatment, but as a first attempt to organize and synthetize the current landscape on this topic, as well as to offer guidance to future research.

Our survey highlights the clear need for standardizing the reporting of HT cases. Due to the rarity of this phenomenon, only detailed descriptions will contribute to the development of robust knowledge on the subject.

## Disclaimer

The views expressed in the submitted article are our own and not an official position of the institution.
